# Early Clinical Experience With Robot-Assisted Electrode Insertion in Cochlear Implantation Using the Otoarm-Otodrive System

**DOI:** 10.1177/19160216261460457

**Published:** 2026-09-08

**Authors:** Kevin Jer V. David, Vincent Y. W. Lin, Joseph M. Chen, Jennifer L. Spiegel, Amy H. Ng, Kari L. Smilsky, Trung N. Le

**Affiliations:** 1 Department of Otolaryngology – Head and Neck Surgery, Sunnybrook Health Sciences Centre, University of Toronto, ON, Canada; 2 Sunnybrook Research Institute, University of Toronto, Toronto, ON, Canada

**Keywords:** cochlear implantation, robotic surgery

## Abstract

**Importance:**

Manual cochlear implant (CI) electrode insertion remains surgeon-dependent and may contribute to intracochlear trauma. Robotic-assisted systems, such as the new Otoarm-Otodrive, offer motorized control at ultra-slow speeds that aim to minimize force fluctuations and pressure changes, potentially improving hearing preservation and surgical consistency.

**Objective:**

To describe the institutional experience, surgical workflow integration, and feasibility of robot-assisted cochlear implantation (CI) using the Otoarm-Otodrive system.

**Design:**

Retrospective descriptive study of 50 consecutive robot-assisted CI cases.

**Setting:**

Tertiary referral otology center in Canada.

**Participants:**

Fifty Adult patients undergoing cochlear implantation without cochlear malformation or ossification using a MED-EL device from January to October 2025.

**Intervention:**

Robot-assisted cochlear electrode array insertion using the Otoarm-Otodrive (MED-EL) system.

**Main Outcome Measures:**

Feasibility (setup and insertion times), insertion depth, intraoperative challenges, and integration into operative workflow.

**Results:**

The additional time for robot setup and alignment averaged 374 seconds. Mean electrode insertion duration, performed at a standardized rate of 0.1 mm/second, was 444 seconds. Postoperative Otoplan analysis in 30 cases demonstrated a mean angular insertion depth of 575°. No major complications such as facial nerve palsy or infections, and no device-related complications occurred. All but 1 case achieved full insertion.

**Conclusions:**

Robot-assisted cochlear electrode insertion using the Otoarm-Otodrive system is safe, reproducible, and easily integrated into standard CI workflow.

**Relevance:**

This first North American experience in using the Otoarm-Otodrive system demonstrates the feasibility of robotic cochlear implant insertion as a step toward achieving atraumatic implantation. Future studies will assess hearing preservation benefits and cost-effectiveness for broader adoption.

## Graphical abstract

**Graphical Abstract:**
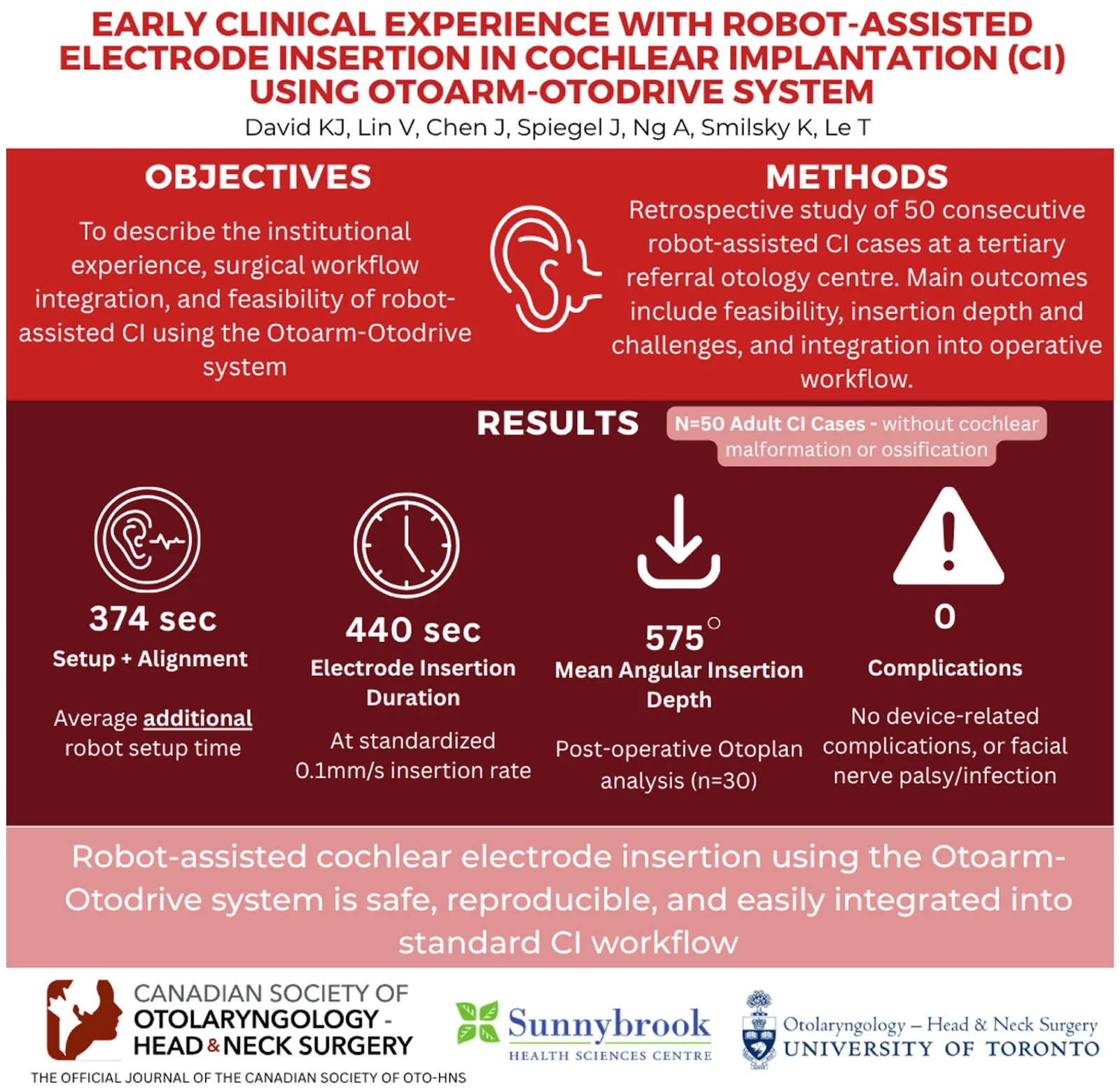
This is a visual representation of the abstract.

## Introduction

Cochlear implantation (CI) has become a cornerstone in the management of severe to profound sensorineural hearing loss, restoring auditory perception by directly stimulating the cochlea and auditory nerve. As indications for implantation have expanded to include individuals with measurable residual hearing, surgical goals have evolved beyond electrode placement toward preserving existing cochlear function. Achieving this requires techniques that minimize mechanical trauma and intracochlear pressure fluctuations during electrode insertion as measures to preserve residual hearing function.^1-3^

Despite advances in electrode design and surgical approaches, variability in hearing preservation outcomes persists. Manual insertions depend heavily on the surgeon’s skill, pace, and tactile feedback. Human factors can lead to unpredictable insertion forces and intracochlear pressure changes, increasing the risk of cochlear trauma and loss of residual hearing.^4-7^

These challenges have prompted the development of robotic systems designed to deliver precise, consistent, and controlled electrode insertions while reducing operator-dependent variability. Among the currently available platforms are the Otoarm-Otodrive (MED-EL, Innsbruck, Austria),^8^ iotaSOFT Insertion System (iotaMotion Inc, MN, USA),^9^ and RobOtol (Collin, France).^10,11^ Preclinical and early clinical investigations have demonstrated that these systems can significantly reduce insertion forces and intracochlear pressure peaks compared with manual techniques, suggesting a potential advantage in minimizing cochlear trauma.^12-14^

The present study focuses on the Otoarm-Otodrive system, which provides motorized, foot-pedal-controlled electrode advancement at ultra-slow speeds while maintaining the surgeon’s control over trajectory and depth. Cadaveric temporal bone experiments using synchrotron phase-contrast imaging have previously shown that this system can minimize damage to intracochlear structures.^15^ However, the safe integration of robotic technology into live otologic surgery requires validation through real-world clinical experience to confirm its feasibility, safety, and efficiency.

In this study, we describe our early institutional experience with robot-assisted cochlear implantation using the Otoarm-Otodrive system, the first clinical series performed in North America. Our objective is to provide a practical guide for incorporating the system into routine clinical practice, including operative setup, positioning, technical execution, and troubleshooting. We also present feasibility data on setup time and insertion parameters across 50 consecutive cases.

## Methodology

### Patient and Electrode Selection

All adult patients undergoing conventional cochlear implantation using a MED-EL device at our institution were eligible for robotic insertion. Standard indications for CI applied and vetted by the cochlear implant team. Patients with cochlear malformations or ossified cochleae were excluded from robotic use, as these cases often require customized electrode, modified surgical protocol, or partial insertions. Pre-operative radiographic analysis using the CI manufacturer software (Otoplan) was done to determine the appropriate electrode length for individualized use.

#### The MED-EL Otoarm-Otodrive System

**Figure 1.**
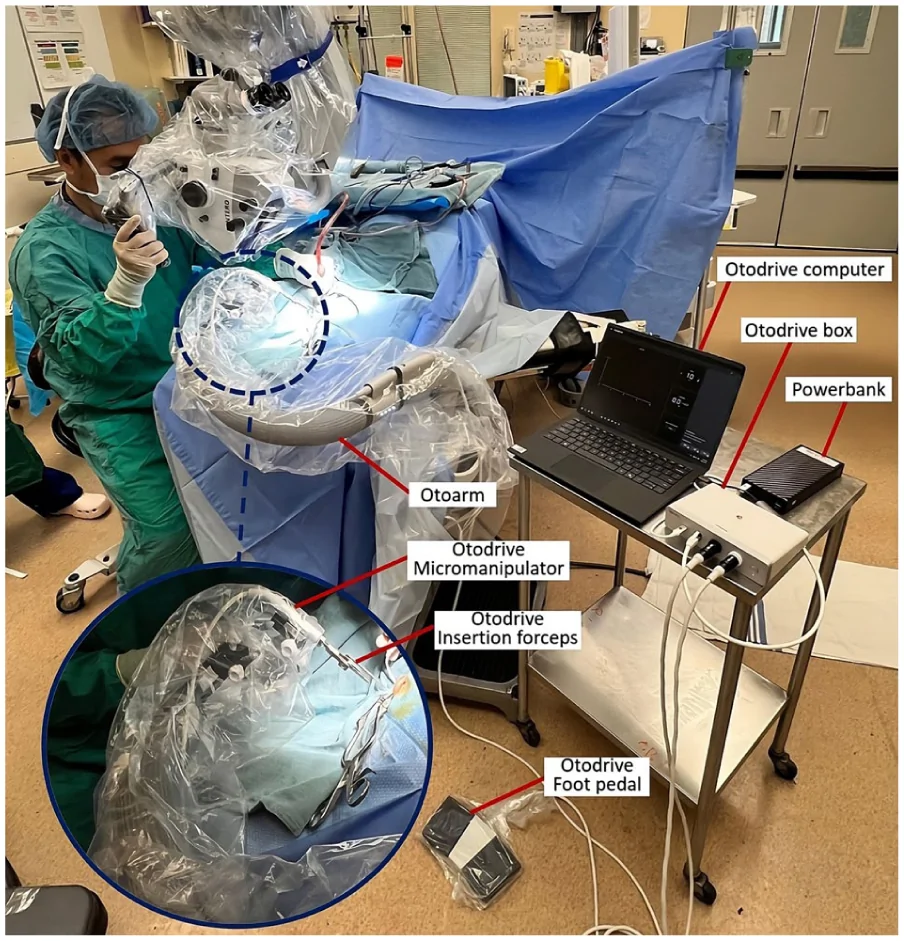
Otoarm-Otodrive parts and setup.

The Otoarm-Otodrive system is composed of a flexible robotic arm (Otoarm), a controllable micromanipulator console for trajectory alignment, and a motorized insertion handpiece (Otodrive) capable of ultra-slow advancement through a footpedal (Figure 1). The entire system is powered by a portable battery pack.

**Figure 2.**
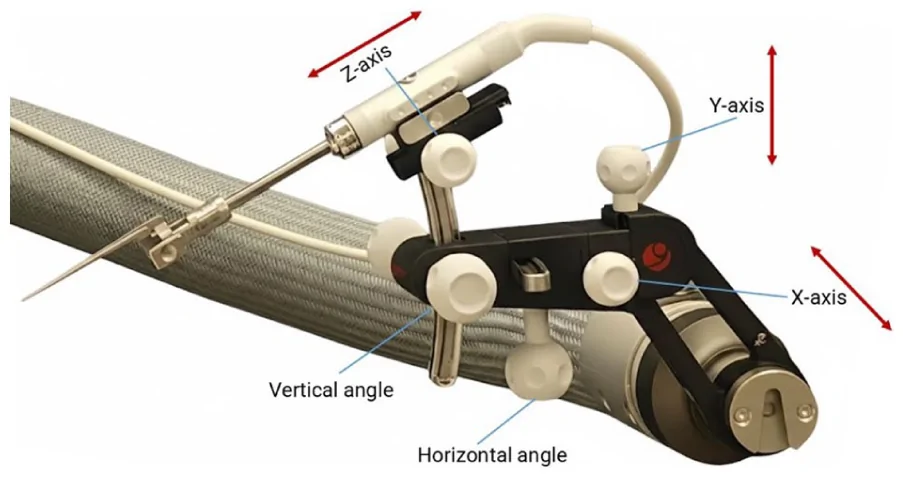
The trajectory of the OTOARM Aligner and insertion forceps can be finely adjusted through the controllable micromanipulator console.

The base of the arm is secured to the surgical bed across the primary surgeon. The locking mechanism is simple and can be clamped onto the headrest side railing. The arm itself extends approximately 1 m around the headrest and can be freely maneuvered in multiple directions before being securely locked into position. At its distal end attaches the controllable micromanipulator console, which allows for manual precise fine-tuning of the array trajectory along the linear (*x*, *y*, *z*) axes as well as angular rotation at the vertical and horizontal axes (Figure 2). A magnetic coupling handpiece holds the robotic insertion forceps, which are linked to a motor (Otodrive) that regulates insertion speed from 0.1 to 1.0 mm/second. Forward and reverse motion along the *z*-axis is activated via a foot pedal, enabling hands-free control during electrode advancement.

### Mastoidectomy and Facial Recess Approach

The patient is positioned supine with the head turned contralaterally, consistent with routine CI surgery. The initial steps of cochlear implantation are similar to conventional CI surgery whereby a post-auricular incision is made followed by developing a wide posteriorly-based Palva flap. A tight submusculoperiosteal flap is prepared and a recess is drilled to accommodate the CI receiver-stimulator and proximal electrode. A canal wall-up mastoidectomy is done to visualize the antrum, lateral semicircular canal and short process of the incus. The incus is then followed to drill into the facial recess while preserving the chorda tympani and the fallopian canal. Posterior tympanotomy is completed with excellent visualization of the stapedial tendon, pyramidal eminence, round window niche, and hypotympanum. The round window bony lip is drilled away to expose the membrane, which is gently and partially opened by lifting the annulus.

#### Setup and Draping of the Otoarm-Otodrive

**Figure 3.**
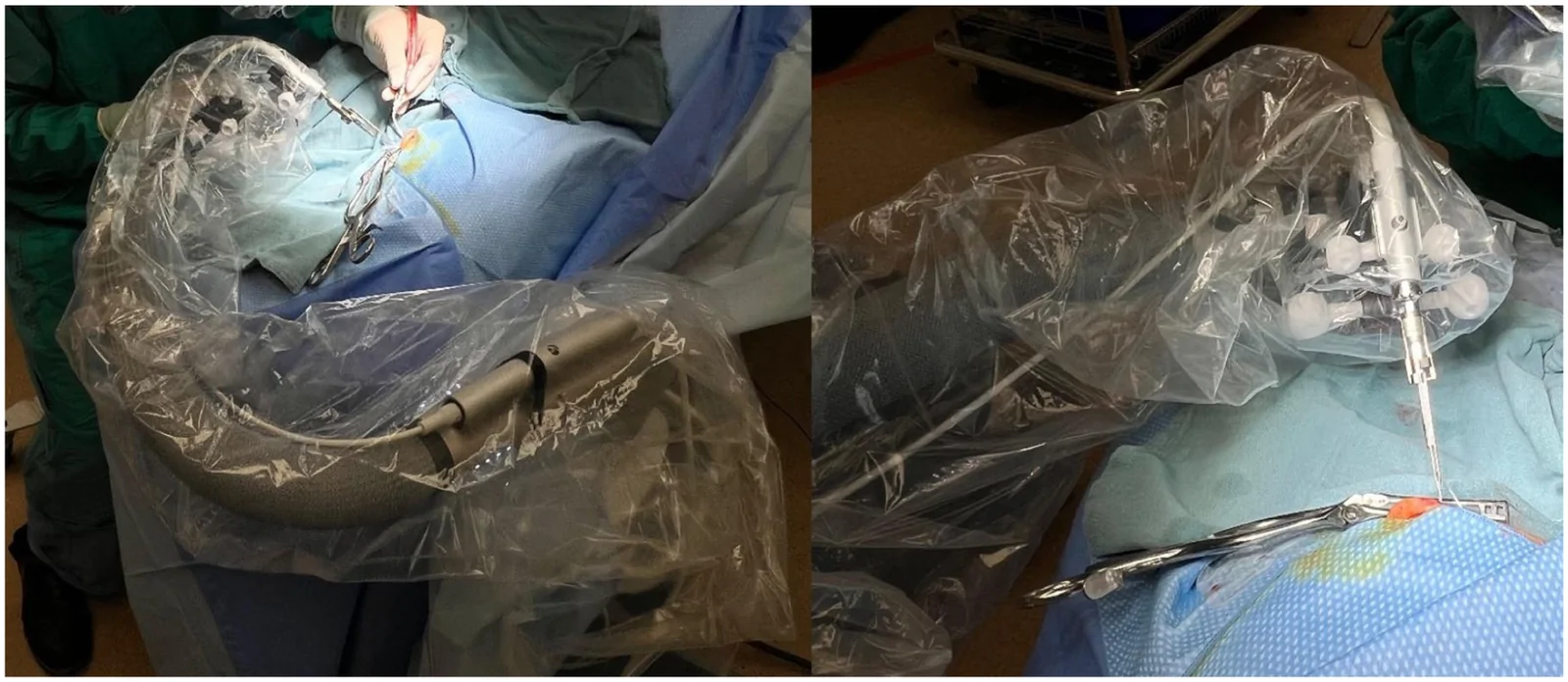
The Otoarm-Otodrive system covered in surgical drapes with the sterile robotic insertion forceps.

In this pilot series, 2 setup sequences were tested. Either the robot was fixed to the surgical bed and draped at the start of the surgery or after drilling the facial recess and the round window niche and prior to electrode insertion. The advantage of setting it up early is a smooth flow in the operating room (OR) and immediate insertion after exposing the round window membrane. However, it occupies space and limits the standing position for the assistant across the surgeon during mastoidectomy. Care must also be taken to prevent hitting the arm throughout the procedure. Our surgeons prefer setting up the arm later, just prior to electrode insertion, because it allows freedom to decide whether robotic insertion is appropriate to the case prior to setting it up. It prevents unnecessarily setting up the robot when the facial recess is very tight or when there is intraoperative suspicion of cochlear fibrosis whereby manual insertion is preferred. Minor disadvantages include the need to lift the surgical drapes in order to hook the arm to the bed headrest railing and care necessary to prevent contamination during setting up the robot after draping. Figure 3 demonstrates the robot covered in a manufactured sterile drape.

### Robotic Arm Alignment

**Figure 4.**
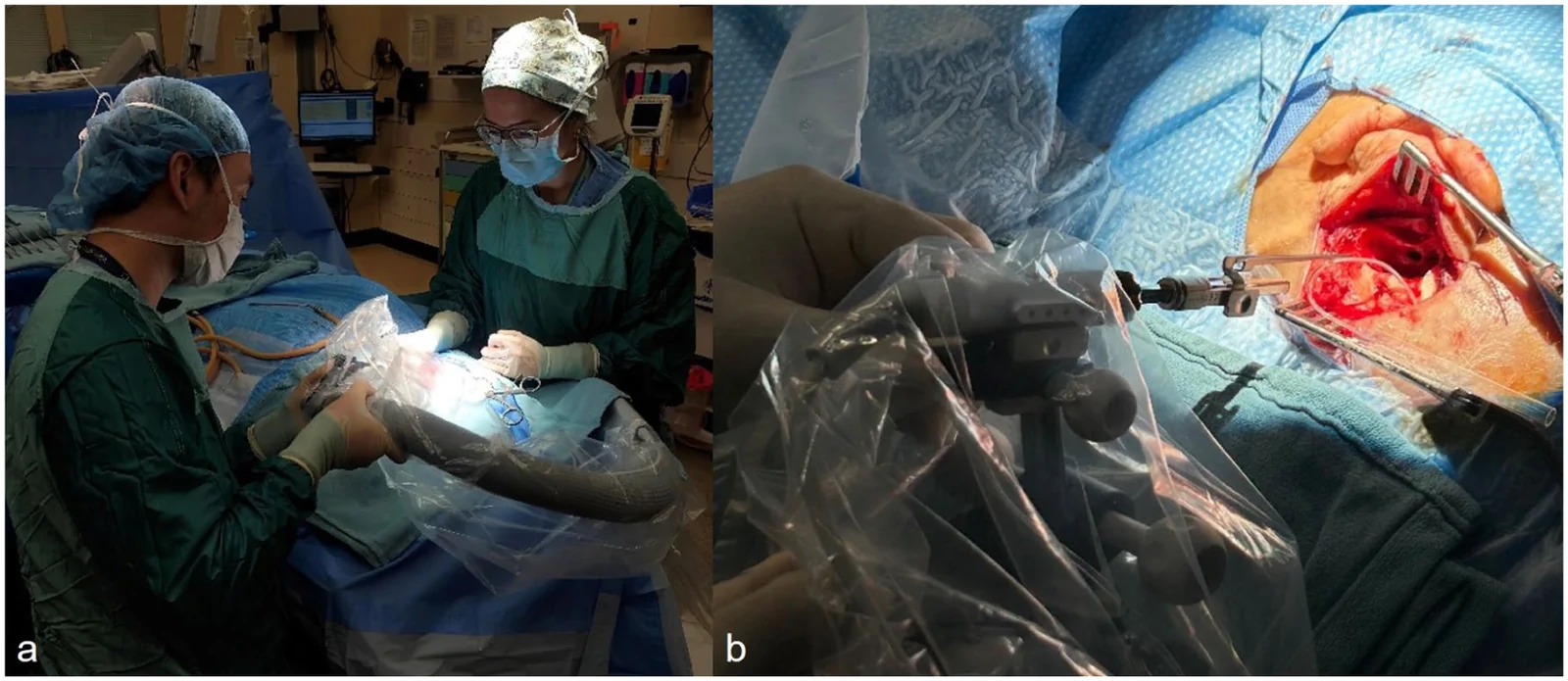
Moving the robotic arm to the surgical field (a). Setting the trajectory toward the facial recess using the fine adjustment knobs (b).

Once the facial recess and round window are exposed, the microscope is temporarily moved away from the field and the flexible robotic arm is brought into position. By pressing a release button, the arm can be freely maneuvered while holding the insertion micromanipulator console. (Figure 4a) The tip of the insertion tool is pointed to the facial recess and grossly aligned to the intended insertion trajectory. Care must be taken during adjustment to ensure that the tip does not hit the fallopian canal or the auditory canal wall. The tip should also be prevented from entering the facial recess as this will limit visualization and cause resistance when decoupling the electrode array after insertion. The robot arm stabilizes at the set position once the maneuver button is released. We recommend manually holding the arm/console 2 to 3 seconds after releasing the button to allow time for the arm to lock in. If any drift occurs, which is usually a drop in the *y*-axis, it is often minor and can be corrected later on with fine adjustment. From our series of cases, the mean time to set up the robot is 135 seconds, which includes draping and mounting the robot arm.

### Fine Adjustment of Trajectory

Once the arm is locked in place, fine adjustment of the trajectory under microscope visualization is done using the controllable micromanipulator console (Figure 4b). The console has 5 knobs which correspond to linear translation in the *x*-*y*-*z* axis, and angular rotation along the horizontal and vertical axis (Figure 2). This step requires familiarity of the knobs in order to quickly set the desired trajectory. Once set, the pedal is stepped on reverse to pull the robotic insertion forceps all the way backward to load the electrode array.

### Electrode Array Mounting

**Figure 5.**
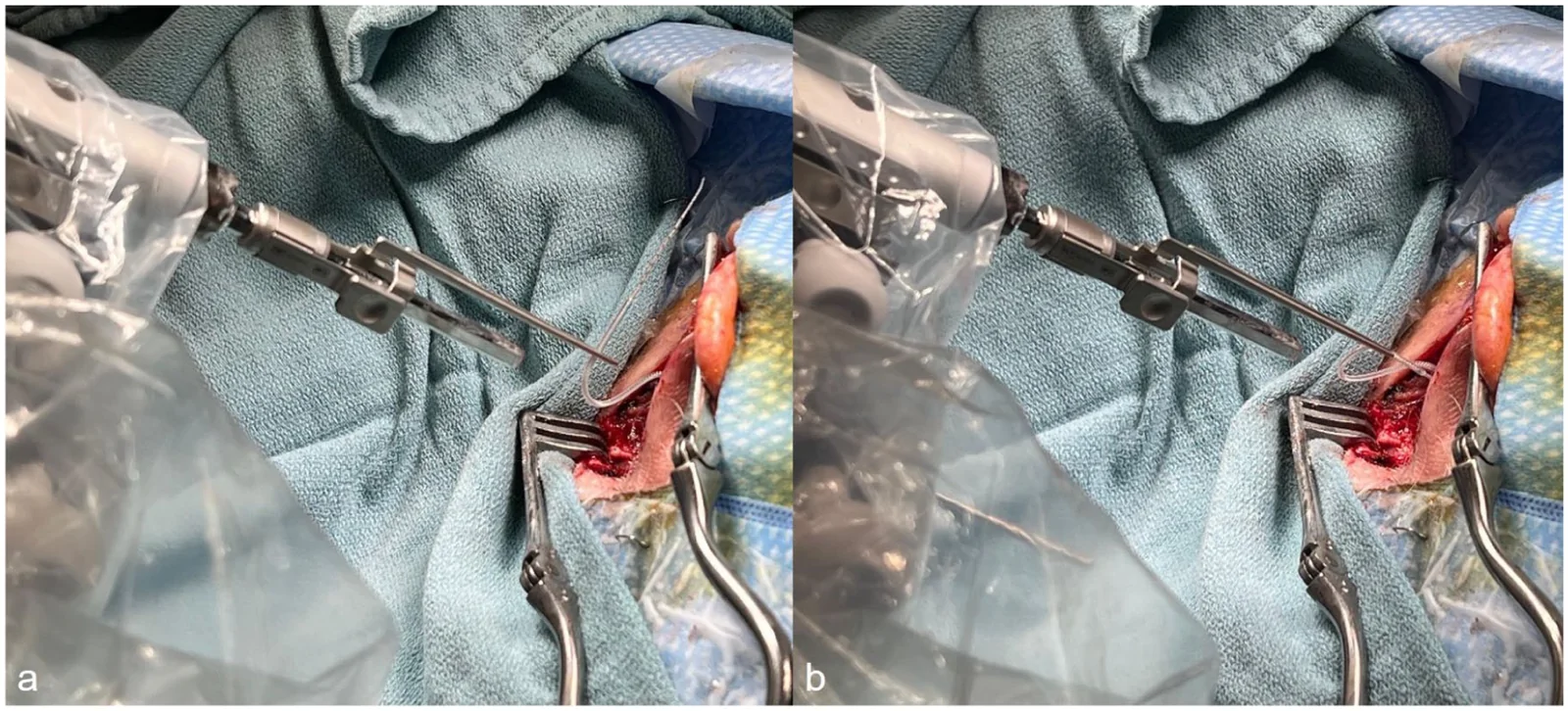
The electrode array is initially mounted perpendicular to the forceps (a) then rotated forward to align with the trajectory (b).

At this point, the electrode array is loaded onto the tip of the retracted robotic insertion forceps located at the tip of the micromanipulator console. The array is initially loaded perpendicular to the forceps and then rotated forward to align with the trajectory (Figure 5). The tip of the forceps is mounted at 10 to 15 mm proximal to the electrode array marker. It must be ensured that the array is locked securely into the forceps and that it is parallel with the trajectory of the forceps to avoid slips and buckling. The tip of the electrode array is then gently adjusted manually to ensure it is inside the facial recess and pointing toward the opened round window. In our series, the average time to finely set the trajectory using the micromanipulator console and point the electrode array tip to the round window is 239 seconds.

### Electrode Insertion

**Figure 6.**
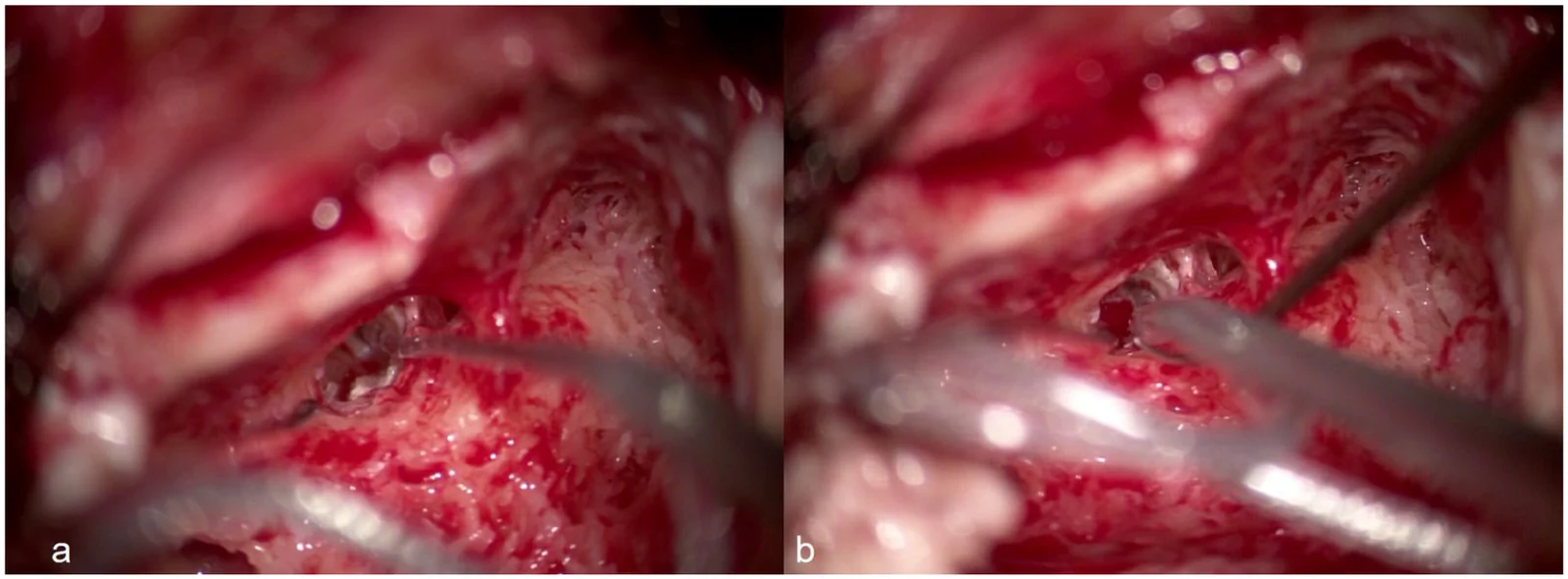
Microscopic view of electrode array insertion using Otodrive (a). Manual intervention to address buckling using Rosen needle (b).

To begin insertion, the electrode array is manually advanced using the *z*-axis knob of the console until the tip barely enters the round window. At this point, the Otodrive motor takes over the insertion at the set speed of 0.1 mm/second using the foot pedal as shown in Figure 6. During advancement, dexamethasone is regularly injected into the facial recess. The surgeon closely monitors for any signs of resistance or electrode buckling and may intervene as needed to adjust the trajectory, pause the insertion, or straighten the electrode (Figure 6b). In many cases, minor buckling automatically corrects itself as the array is advanced at an ultra-slow speed. Frequent manual interventions may negate the constant, low insertion force offered by the automated insertion. Array is advanced until full insertion is achieved, similar to routine CI insertions. In our series, the mean time for electrode insertion from tip to finish is 444 seconds. In some instances, robotic insertion is abandoned for the final electrode contact due to significant buckling. In these cases, insertion of the final electrode contact is completed manually using traditional manual insertion forceps.

### Decoupling

Once full insertion is achieved, a jeweler’s forceps or manual insertion forceps is used to stabilize the electrode while decoupling it from the robotic insertion forceps. Once disconnected, the instrument is pulled back, and the robotic arm is pulled away from the surgical field. Care must be taken to ensure complete decoupling of the forceps and to prevent snagging the electrode during pull-out.

### Final Steps

The final steps are similar to routine CI surgery. Soft tissue is obtained from the periosteum, temporalis fascia or muscle to plug the round window niche. The rest of the electrode array is then secured under the ledge of the mastoid cortex and additionally stabilized using large pieces of gelfoam. The posteriorly-based Palva flap is closed and skin is closed using sutures and surgical staples. Intraoperative X-ray is then done to confirm electrode array placement prior to breaking sterility and dressing. The Otoarm-Otodrive system can be removed easily by unclamping it from the bed railing.

## Challenges and Troubleshooting

- *Limited Facial Recess:* When using the robot, it is preferred to maximize the facial recess exposure to allow for good visualization, room for manipulating tools, and excellent light entry. The fallopian canal is skeletonized, the posterior canal wall is thinned down, and the facial recess is extended inferiorly while preserving the facial nerve and chorda tympani nerve. In cases when the facial recess is anatomically very tight, automated insertion may be abandoned entirely or for inserting the last electrode contacts.
- *Trajectory Misalignment:* Precision is advised when setting the trajectory of the insertion tool, as buckling and manual intervention mid-insertion usually result from poor alignment. We recommend practicing with the console knobs pre-operatively to gain familiarity with the device. A common mistake would be to point the tip toward the round window, but not ensuring the ideal advancement trajectory. The ideal advancement trajectory is the “anterior inferior” direction where the robotic forceps are pointing in the same direction as the electrode toward the basal turn of the cochlea. The micromanipulator console has knobs for vertical and horizontal angulation which maintain the tip in the same position but adjusts the trajectory angle of insertion.
- *Improper Mounting and Unmounting of the Electrode Array.* In our pilot series, we have tried multiple mounting points and recommended holding the electrode 10 to 15 mm from the electrode marker to the tip of the robotic forceps. Mounting it too close to the marker, 5 mm or less, results in premature bumping of the tip of the forceps to the fallopian canal or ear canal, preventing deep automated insertion. Mounting it too far, 20 mm or more, results in less rigidity and more buckling during insertion.

During mounting, we also recommend holding the electrode array perpendicular to the insertion forceps first, then rotating it forward to align with the instrument. Trying to mount it parallel to the forceps may result in twisting of the forceps arms or inadequate hold of the array. Unmounting also requires significant care, since any harsh movement may cause increased intracochlear pressure changes as described in vitro by Cramer et al^16^ and Aebischer et al.^17^ Worse, it could result in partial or full electrode array pull out.

- *Learning Curve:* The whole surgical team, including the nurses and scrub technicians, should be familiarized with the equipment and setup. Simulations and practices are recommended prior to pilot use to prevent delays and errors. Early cases may require prolonged setup times, particularly in setting the electrode trajectory. With experience, its use became faster and more reproducible.
- *Conversion to Manual:* In cases where alignment was unsatisfactory or significant buckling was observed, the approach can be converted to manual insertion without complication. In our experience, it was not uncommon to convert to manual insertion for the last electrode contact. Early conversion to manual insertion decreased with more cases as it often occurred due to correctible reasons such as holding the array too distally or improper mounting of the array.

## Clinical Experience and Feasibility Data

We performed robot-assisted cochlear implantation using the Otoarm-Otodrive system in 50 patients. The electrode arrays used included 42 FLEX28 arrays and 8 FLEXSoft arrays. The patient cohort comprised 14 cases (28%) of single-sided deafness and 36 cases (72%) of bilateral hearing loss, with 7 patients receiving their second cochlear implant. The additional setup time required for robotic use was modest. On average, setting up and moving the robotic arm into the operative field required 135 ± 37 seconds, while mounting the electrode array and setting up the trajectory toward the round window took 239 ± 43 seconds on average. In total, the additional robotic setup time prior to actual array insertion averaged at 374 ± 50 seconds.

Electrode advancement was performed at a standardized rate of 0.1 mm/second, requiring a mean insertion time of 444 ± 75 seconds, with the shortest possible time (at 0.1 mm/second speed without pausing) being 308 seconds. Although the insertion rate is fixed, time varied because of occasional pauses to check and manually adjust the trajectory. The overall mean operative time from skin incision to closure was 83 minutes (range: 70-95 minutes).

Postoperative Otoplan reconstruction and analysis was completed in 33 cases which demonstrated mean angular insertion depth of 575° ± 49° (Flex28: 561° ± 53°, FlexSoft: 602° ± 41°). Based on imaging analysis, full insertion was achieved in all cases except one (97%) which had 1 extracochlear electrode contact at the round window level. No major complications such as infections, and facial nerve palsy occurred and no device-related complications were observed.

From a feasibility standpoint, the robotic system integrated smoothly into the surgical workflow. Although early cases required longer alignment times, efficiency improved after approximately 5 to 10 cases.

Hearing preservation and speech perception outcomes are not yet available, as meaningful audiometric analysis requires at least 12 months. These data will be reported in future studies.

## Discussion

Our early institutional experience, encompassing 50 robotic-assisted cochlear implantation (CI) surgeries, suggests that the Otoarm-Otodrive system can be implemented in the OR with a favorable safety profile. A crucial aspect of adopting new technology is the impact on OR efficiency. The additional setup time was minimal, measured at approximately 6 minutes. This finding is comparable with previous studies including a pilot use of the RobOtol system in China, similarly documenting a 6 minute setup time for insertion.^18^ Furthermore, a survey of 16 surgeons in the United States noted that the total OR time added by robotic assistance in CI was between 5 and 15 minutes, with respondents reporting increased comfort after as few as 5 cases.^19^

Our series has also shown that robotic assistance can be integrated in the OR without alteration of the established CI workflow. Standard mastoidectomy and posterior tympanotomy are performed manually, and the robot is introduced only prior to the critical electrode insertion phase. This simplicity of clamping the system on and off the surgical bed railing facilitates clinical adoption and lowers the barrier for surgeons already proficient in conventional techniques.

The fundamental clinical benefit of the Otoarm-Otodrive is its capacity to mitigate insertion-related trauma. Previous cadaveric and experimental studies have consistently demonstrated that this system reduces insertion forces, intracochlear pressure fluctuations and trauma compared to manual insertions.^12,15^ These preclinical findings strongly suggest a mechanism for improved hearing preservation. Our clinical series complements this research by confirming that this controlled, precision-driven insertion is feasible in the dynamic environment of the OR.

While feasibility is confirmed, the critical question remains whether this mechanical advantage translates into improved hearing outcomes, particularly for patients with residual hearing. One study utilizing the RobOtol system in 24 insertions documented better hearing preservation in the low frequencies (250-1000 Hz) compared to manual insertion.^20^ To fully validate the clinical utility of the Otoarm-Otodrive, long-term follow-up of audiometric data and a dedicated cohort of patients with residual hearing is essential to determine if similar objective hearing preservation can be achieved.

Our experience represents early institutional use, and broader multicenter data are needed to validate the generalizability of these initial findings. While cost and accessibility remain potential challenges for widespread adoption, robotic-assisted insertion holds promise as a valuable adjunct in CI surgery, particularly for patients in whom atraumatic electrode placement is critical for optimal long-term functional results.

## Conclusion

Our initial institutional experience indicates that robotic-assisted cochlear electrode array insertion with the Otoarm-Otodrive platform is technically feasible and carries a favorable safety profile. Our experience with 50 cases demonstrates successful insertion, no complications, and manageable setup times. The system integrates seamlessly into routine CI surgery and offers a promising platform for atraumatic implantation. Ongoing follow-up will clarify its role in optimizing hearing preservation outcomes.

## Key Messages

- The Otoarm-Otodrive system is safe, reproducible, and easily integrated into the standard cochlear implantation workflow, adding an average of 6 minutes to the operative time.
- Across 50 cases, the robot-assisted technique demonstrated no major complications and achieved full electrode insertion in nearly every case.
- This study establishes the first published North American clinical experience, providing a practical, data-supported guide for other centers to adopt precision-controlled robotic insertion as a step toward reducing intracochlear trauma.
